# Perturbed adipose tissue hydrogen peroxide metabolism in centrally obese men: Association with insulin resistance

**DOI:** 10.1371/journal.pone.0177268

**Published:** 2017-05-18

**Authors:** May G. Akl, Eman Fawzy, Maha Deif, Ayman Farouk, Amany K. Elshorbagy

**Affiliations:** 1 Department of Physiology, Faculty of Medicine, University of Alexandria, Alexandria, Egypt; 2 Department of Clinical and Experimental Surgery, Medical Research Institute, University of Alexandria, Alexandria, Egypt; The Ohio State University, UNITED STATES

## Abstract

**Objective:**

Although adipose tissue hydrogen peroxide (H_2_O_2_) and its metabolizing enzymes have been linked to obesity and insulin resistance in animal studies, this relation remains to be evaluated in humans.

**Methods:**

Non-diabetic men (N = 43, median age, 49 (37, 54 y)) undergoing abdominal surgeries were studied. Participants were classified by body mass index (BMI) into normal-weight (N = 19), or overweight/obese (Ow/Ob; BMI ≥25; N = 24). Centrally obese men were identified by waist-height ratio ≥0.5. H_2_O_2_ and activities of superoxide dismutase, catalase and glutathione peroxidase enzymes were assayed in subcutaneous fat samples, and visceral fat (available from N = 33), and their associations with anthropometric parameters, fasting serum lipids, and the homeostasis model of insulin resistance (HOMA-IR) were tested using correlations and multivariate linear regression.

**Results:**

H_2_O_2_ concentrations and catalase activity were increased in visceral fat from Ow/Ob men, compared to normal-weight subjects (+32%, P = 0.038 and +51%, P = 0.043 respectively). Centrally obese subjects had >2-fold higher superoxide dismutase activity (P = 0.005), 46% higher H_2_O_2_ (P = 0.028), and 89% higher catalase activity (P = 0.009) in visceral fat, compared to lean subjects. Central obesity did not alter these markers in subcutaneous fat, apart from a 50% increase in catalase, and did not affect glutathione peroxidase in either fat depot. H_2_O_2_ in visceral fat positively correlated with insulin resistance (r = 0.40, P = 0.032). Catalase activity in visceral fat was an independent determinant of HOMA-IR, explaining ~18% of the variance (*ß* = 0.42, P = 0.016), after adjustment for age and BMI.

**Conclusion:**

These findings suggest that adipose tissue catalase shows compensatory up-regulation in response to obesity-induced H_2_O_2_ accumulation, and that perturbed H_2_O_2_ metabolism in visceral fat is linked to insulin resistance in obese humans.

## Introduction

According to the WHO, 42% of the world population were overweight or obese in 2014 [1]. Obesity is a major public health problem that predisposes to chronic morbidity and is a drain on healthcare resources worldwide [2]. One of the most common complications of obesity is insulin resistance, which culminates in type 2 diabetes [3]. An understanding of how obesity predisposes to insulin resistance is instrumental in devising therapies that prevent insulin resistance and hence diabetes.

Oxidative stress has received increasing attention as a potential mechanism linking obesity to associated morbidities including insulin resistance, diabetes, dyslipidemia and cardiovascular disease [4, 5]. Reactive oxygen species (ROS) such as superoxide (O^2•-^), hydrogen peroxide (H_2_O_2_), and hydroxyl radical (OH^•^) are normally maintained at non-toxic levels through an efficient antioxidant defense system. Superoxide formed inside the mitochondria is rapidly reduced to H_2_O_2_ by superoxide dismutase enzyme [6]. Glutathione peroxidase then uses glutathione to degrade H_2_O_2_, forming glutathione disulfide and water [7]. Catalase also provides an efficient mechanism of scavenging H_2_O_2_ as it decomposes H_2_O_2_ without consumption of reducing equivalents, producing O_2_ and water. H_2_O_2_ is an important source of oxidative stress because it is a small, diffusible molecule that is relatively stable under physiological conditions [8].

The diverse health effects of genetic deletion or overexpression of antioxidant enzymes have been recently reviewed [9]. Consistent with the body of literature linking obesity with oxidative stress, glutathione peroxidase and catalase enzyme activity and mRNA expression were shown to be lower in adipose tissue of obese animals [10, 11]. In addition H_2_O_2_ production was increased in adipose tissue of obese mice [12]. H_2_O_2_ formation was also increased in skeletal muscle of obese, insulin resistant humans, and in subjects fed a high-fat diet, while blocking H_2_O_2_ generation in mice fed a high fat diet preserved insulin sensitivity [13]. Moreover, erythrocyte catalase activity was lower in children with insulin resistance and obesity [14, 15]. Collectively these studies suggest that obesity triggers oxidative stress and that oxidative stress is directly linked to insulin resistance.

The association of H_2_O_2_ in adipose tissue with insulin resistance, however, has not been studied in humans. Adipose tissue is unique in that, paradoxically, oxidants mimic insulin action on glucose transport in adipose cells [16]. H_2_O_2_ in small concentrations facilitates insulin signaling via oxidative inhibition of protein tyrosine phosphatases that inhibit insulin action [17]. In line with this, glutathione peroxidase knockout mice are protected against high-fat diet-induced insulin resistance [18, 19]. It is not known whether glutathione peroxidase and catalase activities are increased or decreased in adipose tissue from obese humans, and how this links to H_2_O_2_ concentrations and peripheral insulin sensitivity.

In the present study, we examined whether human obesity is associated with changes in H_2_O_2_ metabolism in the visceral and subcutaneous fat depots, and whether these changes are linked to insulin resistance.

## Methods

### Study participants

The study was approved by the Ethics Committee of the Faculty of Medicine, Alexandria University. Study participants were recruited among patients undergoing elective abdominal surgeries at the Department of Experimental and Clinical Surgery, Medical Research Institute, University of Alexandria, and at the General Surgery Department at the Alexandria University Hospital. The surgeries included inguinal herniotomy, closure of abdominal incisional hernias, cholecystectomy, and excision of benign lipomas. All subjects had the study explained to them and provided written informed consent before their inclusion in the study.

#### Inclusion and exclusion criteria

Sedentary men, aged 20-65y were recruited, without regard to weight status, but with the aim of including a wide range of BMI to enable group comparisons based on BMI cutoffs. Exclusion criteria comprised the presence of type 1 or type 2 diabetes, chronic liver or renal dysfunction, cancer, cachexia, use of lipid lowering drugs, as well as gain or loss of > 5% of body weight over the preceding 3 months. Only men were included in the study to avoid the potential influences of hormonal changes during different phases of the menstrual cycle on redox state [20].

### Medical history and anthropometric measurements

Information was collected on patients’ lifestyle (exercise and cigarette smoking) and medical history, family history of cardiovascular disease and diabetes mellitus, and the type of surgery for which the patient was admitted. Smoking was coded as never-smoker, current smoker, or ex-smoker.

Pulse and supine blood pressure, and anthropometric parameters were recorded on the morning of the surgery. Body weight and height were measured to the nearest 0.5 kg and 0.5 cm respectively. Weight was determined using a standard scale with the subjects barefoot and wearing a surgical gown. Height was measured using a wall-mounted stadiometer. Body mass index (BMI) was calculated as weight (kg) /height squared (m^2^). Waist circumference was measured with a non-stretchable measuring tape, at a level midway between the lower costal margin and the iliac crest. Waist and waist-height ratio were used as indices of central adiposity [21].

#### Classification of study participants according to anthropometric cutoffs

Study participants were classified by BMI into normal-weight (BMI <25; N = 19), and overweight/obese (Ow/Ob; BMI ≥25; N = 24).). Study participants were also classified according to their waist-height ratio into 2 groups: a centrally obese group (waist-height ratio ≥0.5, N = 30, including 23 with visceral fat data) and a lean group (ratio <0.5, N = 13, including 10 with visceral fat data). This classification was based on reports that a ratio of 0.5 or higher is associated with cardio-metabolic risk factors including hypertension, type 2 diabetes, dyslipidemia, metabolic syndrome and cardiovascular risks [22–24].

### Sampling

#### Serum samples

Venous blood was collected on the morning of the surgery after an overnight fast. The samples were withdrawn before induction of anesthesia, and transported on ice to the laboratory. Clotted samples were centrifuged, (10,000 x g at 4°C for 4 minutes), and the resultant serum was aliquoted and stored at -80°C until analysis.

#### Adipose tissue samples

Superficial subcutaneous adipose tissue was collected at the site of the abdominal surgical incision. In cases where the type of surgery entailed a peritoneal incision, omental (visceral) fat samples were collected immediately after the incision was made. Samples were instantly snap-frozen in liquid nitrogen and stored at -80°C until analysis [25].

### Serum clinical biochemistry and peripheral insulin sensitivity

Fasting serum total cholesterol, LDL cholesterol (LDL-C), HDL-C, triglycerides and glucose were measured by calorimetric assays on a Stat Fax 1904 Plus spectrometer (Awareness Technology, Inc., Palm City, Florida, USA), with absorbance at 474–505 nm. Fasting serum insulin was assessed by an enzyme-linked immunosorbent assay using an Insulin Quantitative Test Kit (Immunospec Corporation, CA, USA) according to the manufacturer’s instructions. Homeostasis model assessment-insulin resistance index (HOMA-IR) was calculated using the formula: HOMA-IR = [glucose (mg/dL) * insulin (μU/mL)/405], and used as an index of insulin resistance [26].

### Assessment of adipose tissue hydrogen peroxide metabolism

Visceral and subcutaneous adipose tissue samples were homogenized in cold buffer (50 mM phosphate-buffer, pH 7.0, containing 5 mM EDTA), and then centrifuged at 10,000 x g at 4°C for 10 minutes. An aliquot from the resultant supernatants, and from the serum samples, was treated by 2 M perchloric acid and then neutralized with 2 M KOH for measurement of H_2_O_2_ concentrations.

#### Hydrogen peroxide concentrations

A Stat Fax 1904 Plus spectrometer (Awareness Technology, Inc., Palm City, Florida, USA) was used in all assays of H_2_O_2_ and its metabolizing enzymes. H_2_O_2_ concentrations were determined in deproteinized serum and adipose tissue samples using a previously described colorimetric method [27], with slight modifications. H_2_O_2_, in presence of Horse Radish Peroxidase, reacts with 3, 5-dichloro-2-hydroxy-benzensulfonic (DHBS) acid and 4-amino-phenazone to generate a chromophore that is detected at an absorbance of 510 nm. The intra-assay and inter-assay coefficients of variation (CV’s) were 4% and 8%, respectively.

#### Glutathione peroxidase enzyme activity

The tissue activity of the H_2_O_2_ metabolizing enzyme glutathione peroxidase was determined using glutathione reductase and then measuring the rate of NADPH oxidation at an absorbance of 340 nm using H_2_O_2_ as the substrate [28]. This assay had intra-assay and inter-assay CV’s of 6% and 7%, respectively.

#### Catalase activity

Activity of the H_2_O_2_ catabolizing enzyme catalase was measured as described previously [29]. H_2_O_2_ reacts with a known quantity of catalase. After adding a catalase inhibitor, the reaction is stopped after one minute. The remaining H_2_O_2_ reacts with 3, 5-dichloro-2-hydroxybenzene sulfonic acid (DHBS) and 4-aminophenazone (AAP) to form a chromophore with color intensity inversely proportional to catalase activity. Absorbance was read at 510 nm against a standard blank [29]. The intra-assay and the inter-assay CV’s were 4% and 7%, respectively.

#### Superoxide dismutase activity

Superoxide dismutase activity was measured calorimetrically according to the method of Nishikimi et al [30]. The assay depends on the ability of the superoxide dismutase to inhibit the phenazine methosulphate-mediated reduction of nitroblue tetrazolium dye, with absorbance read at 510 nm. This assay had intra-assay and inter-assay CV’s of 3% and 4%, respectively.

### Statistical analysis

The Kolmogorov–Smirnov test was used to determine the distribution of the data. For skewed variables, log-transformations or non-parametric analysis were used as appropriate. Subject characteristics are presented as median, 25^th^ and 75^th^ percentiles and the groups were compared by Mann-Whitney *U* test. H_2_O_2_ concentration and activity of its metabolizing enzymes were compared in the visceral versus subcutaneous fat compartments by Wilcoxon signed rank test. Associations of interest were evaluated using Spearman or Pearson correlations as appropriate. Multivariate linear regression was used to investigate whether catalase, superoxide dismutase or H_2_O_2_ were independent predictors of HOMA-IR, after adjustment for age and BMI. Separate models were fitted for each parameter, due to strong collinearity among the predictor variables. PASW Statistics for Windows (20.0; SPSS Inc., Chicago, IL, USA) and GraphPad Prism (version 6.0f for Windows) were used for analysis and presentation of data. All tests were two-tailed and P <0.05 was considered significant.

## Results

### Characteristics of the study participants

Study participants included 43 middle-aged men, with median age 49 y (25^th^, 75^th^ percentiles: 37, 54 y), and a median BMI of 25.4 (22.5, 28.6) kg/m^2^ (Table 1). Five individuals (including 3 in the Ow/Ob group) were on anti-hypertensive medication. The Ow/Ob group (N = 24) had a higher waist circumference and waist-height ratio compared to normal-weight subjects (N = 19). However, the two groups did not differ significantly in age, the proportion of current smokers (just over 50% of each group), or waist-hip ratio. Fasting serum glucose and lipids also did not differ significantly between the groups, but the Ow/Ob group had a higher serum insulin concentration (14.6 (10.5, 22.2) mU/L) compared to normal-weight subjects (9.9 (7, 12.1) mU/L, P = 0.006), and also higher HOMA-IR (P = 0.014) (Table 1).

**Table 1:** Characteristics of the study population ^1^.

	Total population N = 43	Normal-weight N = 19	OW/Ob N = 24	*P value*
Age (y)	49 (37–54)	45 (36–56)	50 (40–54)	*0*.*38*
Weight (Kg)	77 (67–85)	65 (55–70)	84 (79–91)	*<0*.*001*
BMI (Kg/m2)	25.4 (22.5–28.6)	22.4 (20.6–24)	28.2 (26.1–30.1)	*<0*.*001*
Waist (cm)	96 (83–102)	82 (70–90)	101 (96–104)	*<0*.*001*
WHR	0.93 (0.88–0.97)	0.91 (0.83–0.98)	0.94 (0.91–0.97)	*0*.*30*
WHtR	0.55 (0.49–0.60)	0.47 (0.42–0.53)	0.59 (0.55–0.63)	*<0*.*001*
SBP^2^(mmHg)	120 (110–130)	120(110–135)	120 (110–130)	*0*.*97*
DBP (mmHg)	80 (70–90)	80 (70–90)	80 (70–80)	*0*.*28*
***Fasting serum parameters***				
Cholesterol (mg/dl)	183 (163–208)	178 (145.8–193.8)	184.3 (170–212.7)	*0*.*16*
LDL-C (mg/dl)	114 (89–140)	108 (69–133)	115 (108–144)	*0*.*092*
HDL-C (mg/dl)	39.7 (33.2–50.8)	39.5 (35.1–47.8)	40.1 (32.6–50)	*0*.*98*
Triglycerides (mg/dl)	117 (97–146)	103.9 (93–128)	121 (100–155)	*0*.*12*
Glucose (mg/dl)	108 (101–125)	106 (101–123)	115 (99–128)	*0*.*35*
Insulin (mU/L)	12.0 (8.5–17.3)	9.9 (7–12.1)	14.6 (10.5–22.2)	*0*.*006*
HOMA-IR	3.19 (2.44–4.41)	2.88 (1.76–3.75)	3.92 (2.81–6.34)	*0*.*014*

^(1)^ Data are presented as median (25^th^, 75^th^ percentiles) and groups are compared by Mann Whitney *U* test

^(2)^ DBP, Diastolic blood pressure; OW/Ob, Overweight/obese; SBP, Systolic blood pressure; WHR, Waist-hip ratio; WHtR, Waist-height ratio.

Smokers and non-smokers did not differ in anthropometric parameters, HOMA-IR or H_2_O_2_ metabolism (Table 1 in S1 File).

### Hydrogen peroxide metabolism in the subcutaneous compared to visceral fat depots

In the total population, compared to visceral fat, subcutaneous fat had more than 2-fold higher levels of H_2_O_2_ (P <0.001), and 30% lower catalase activity (P = 0.059). No significant differences were noted in superoxide dismutase or glutathione peroxidase activities (Table 2 in S2 File).

### Hydrogen peroxide metabolism in normal weight vs. overweight/ obese subjects

Visceral adipose tissue from Ow/Ob subjects had significantly higher concentrations of H_2_O_2_ (0.91 (0.77, 1.00) mmol/g), compared to lean individuals (0.69 (0.28, 0.82) mmol/g, P = 0.038; Table 2). The increased H_2_O_2_ concentrations appeared to be linked to increased production, since the activity of superoxide dismutase enzyme showed a trend towards an increase (+28%; P = 0.096). A possibly compensatory 51% increase in activity of the H_2_O_2_ catabolizing enzyme catalase was also observed (P = 0.043). However, no difference was observed for glutathione peroxidase activity. In contrast to visceral fat, none of these parameters differed by BMI category in subcutaneous adipose tissue (Table 2), and serum H_2_O_2_ concentrations were not different across the groups.

**Table 2:** Hydrogen peroxide metabolism in normal weight and overweight/obese men^1^.

	Normal weight N = 19	OW/Obese men N = 24	*P value*
**Serum H**_**2**_**O**_**2**_ (μmol/L)	55 (47–63)	53 (48–62)	*0*.*96*
***Visceral fat parameters***			
**H**_**2**_**O**_**2**_ (mmol/g tissue)	0.69 (0.28–0.82)	0.91(0.77–1.00)	*0*.*038*
**Superoxide dismutase** (μmol/ min/ mg protein)	22.0 (8.6–41.9)	28.3 (19.2–50.8)	*0*.*096*
**Catalase** (nmol/ min/ mg protein)	35.3 (22.9–52.8)	52.7 (32.7–78.6)	*0*.*043*
**Glutathione peroxidase** (nmol/ min/ mg protein)	1.19 (0.83–1.36)	1.02 (0.89–1.62)	*0*.*67*
***Subcutaneous fat parameters***			
**H**_**2**_**O**_**2**_ (mmol/ g tissue)	1.72 (0.93–1.91)	1.75 (0.99–1.90)	*0*.*71*
**Superoxide dismutase** (μmol/ min/ mg protein)	18.1 (12.9–30.6)	22.8 (12.5–45.6)	*0*.*53*
**Catalase** (nmol/ min/ mg protein)	26.7 (20.3–37.2)	35.6 (23.4–53.5)	*0*.*32*
**Glutathione peroxidase** (nmol/ min/ mg protein)	1.29 (0.87–1.67)	0.89 (0.81–1.50)	*0*.*35*

^(1)^ Data are presented as median (25^th^, 75^th^ percentiles) and groups are compared by Mann Whitney *U* test. N = 43 for serum and subcutaneous fat parameters, and N = 33 for visceral fat parameters. OW, overweight.

### Hydrogen peroxide metabolism according to waist-height ratio cutoffs

**Fig 1:**
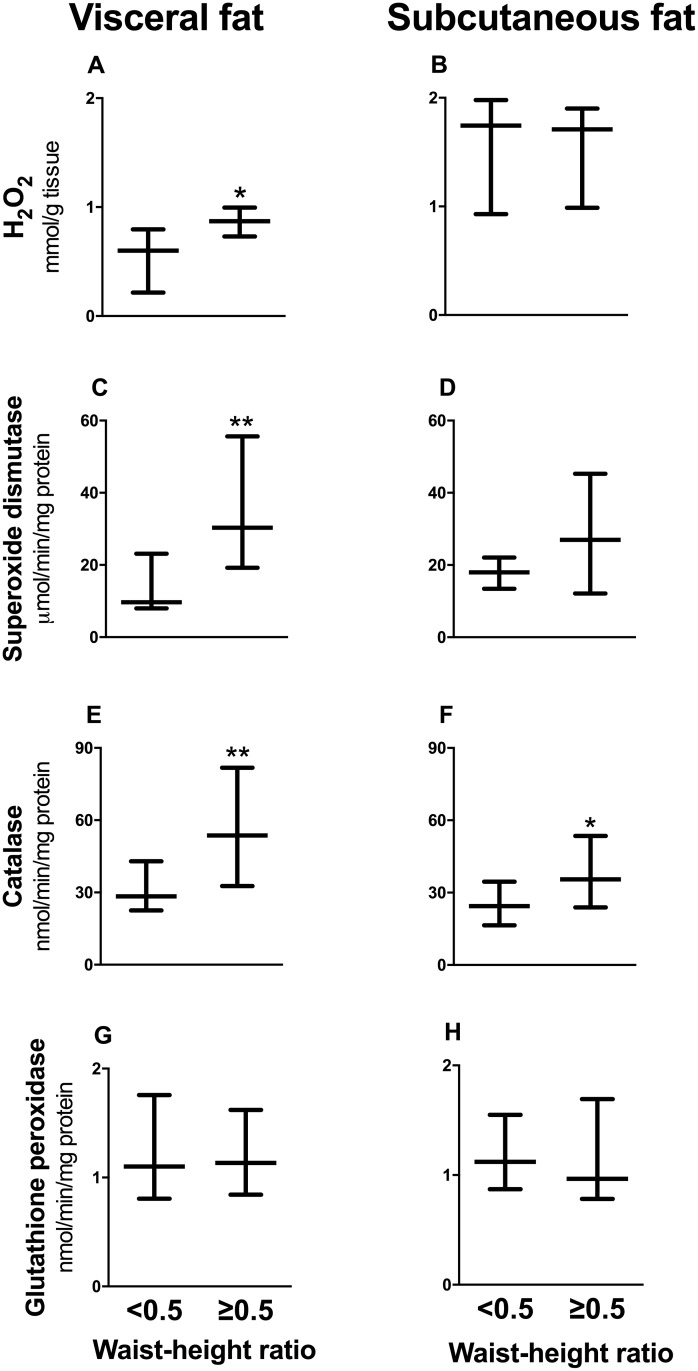
H_2_O_2_ metabolism in visceral (left panel) and subcutaneous (right panel) adipose tissue from centrally obese (waist-height ratio ≥0.5, N = 30) and lean (waist-height ratio <0.5, N = 13) individuals. *A*, *B*, higher H_2_O_2_ levels in visceral but not in subcutaneous adipose tissue of centrally obese individuals. *C*, *D*, Superoxide dismutase activity in visceral but not subcutaneous fat is higher in central obesity subjects. *E*, *F*, Visceral and subcutaneous fat catalase activities are higher in subjects with central obesity. *G*, *H*, No difference between the lean and centrally obese subjects in glutathione peroxidase activity in either fat depot. Values represent medians and 25^th^ and 75^th^ percentiles, *P < 0.05, **P <0.01, Mann Whitney *U* test.

Study participants were also classified according to a waist-height ratio cutoff of 0.5 into 2 groups: a centrally obese group (N = 30), which included 96% of OW/Ob subjects and 37% of normal weight subjects, and a lean group (N = 13). As shown in Fig 1, H_2_O_2_ in the visceral, but not the subcutaneous, fat depot was 24% higher in the centrally obese group compared to the lean group. Centrally obese subjects also had approximately 3-fold and 2-fold higher median superoxide dismutase and catalase activities respectively in visceral adipose tissue, compared to lean individuals. In contrast, no differences were observed in subcutaneous fat apart from a 50% higher catalase activity. Glutathione peroxidase activity was not altered by central obesity in either fat depot.

### Associations of hydrogen peroxide metabolic parameters with central adiposity, lipidemia and insulin resistance

In the total study population, H_2_O_2_ concentrations in visceral fat showed strong positive correlations with BMI, waist, hip circumference and serum triglycerides (r = 0.39–0.51, P = 0.032–0.004; Table 3). Visceral fat superoxide dismutase and catalase activities showed similar positive associations with anthropometric measures. In contrast, H_2_O_2_ and its metabolizing enzymes in the subcutaneous fat depot were not associated with anthropometric measures or serum lipids, apart from a positive correlation of catalase activity (r = 0.30) with waist circumference. Serum H_2_O_2_ concentrations increased with age, but did not correlate with adiposity parameters (Table 3).

**Table 3:** Correlation of age and anthropometric and biochemical measures with markers of hydrogenperoxide metabolism^1^.

	Age	BMI	Waist	Hip	Glucose	Insulin	Triglycerides	Cholesterol
**Serum H**_**2**_**O**_**2**_	0.34*	-0.01	-0.15	-0.25	-0.02	-0.15	0.11	0.01
***Visceral fat parameters***								
**H**_**2**_**O**_**2**_	-0.04	0.42*	0.39*	0.51**	0.18	0.40*	0.42*	0.14
**Superoxide dismutase**	0.14	0.31^2^	0.35*	0.38*	0.30	0.24	0.19	-0.15
**Catalase**	0.23	0.38*	0.44*	0.43*	0.38*	0.42*	0.23	0.00
**Glutathione peroxidase**	0.18	0.10	0.19	0.07	0.2	0.22	0.09	-0.17
***Subcutaneous fat parameters***								
**H**_**2**_**O**_**2**_	0.06	0.02	-0.13	-0.17	-0.06	-0.12	0.13	0.13
**Superoxide dismutase**	0.09	0.22	0.18	0.14	0.03	0.09	-0.02	0.17
**Catalase**	0.06	0.21	0.30*	0.13	0.04	0.01	-0.04	0.00
**Glutathione peroxidase**	0.11	-0.12	-0.04	-0.05	0.25	0.04	0.10	-0.13

^(1)^ Spearman rank correlation coefficients. Significant correlations are depicted in bold font; * P < 0.05, ** P < 0.01. N = 43 for serum and subcutaneous fat measures, and N = 33 for visceral fat parameters.

^(2)^ P = 0.074

**Fig 2:**
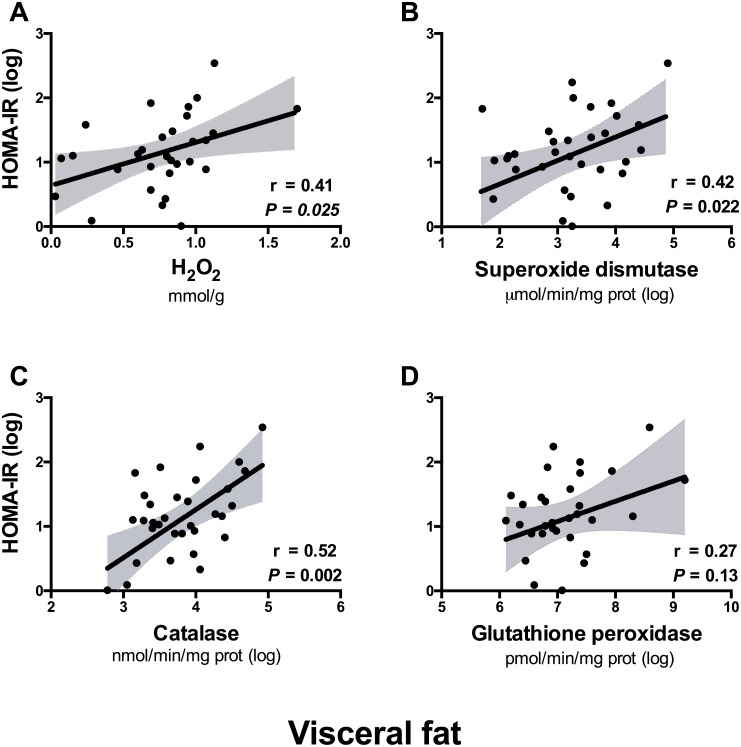
*A*, *B*, *C*, *D* Scatter plots for the associations of H_2_O_2_ *(A)* and activity of its metabolizing enzymes *(B-D)* in visceral adipose tissue with the homeostasis model of insulin resistance (HOMA-IR) in the total population (N = 33). Each plot shows the best-fit linear regression line and 95% confidence intervals, with Pearson correlation coefficients. All parameters are log-transformed apart from H_2_O_2_ concentrations.

**Fig 3:**
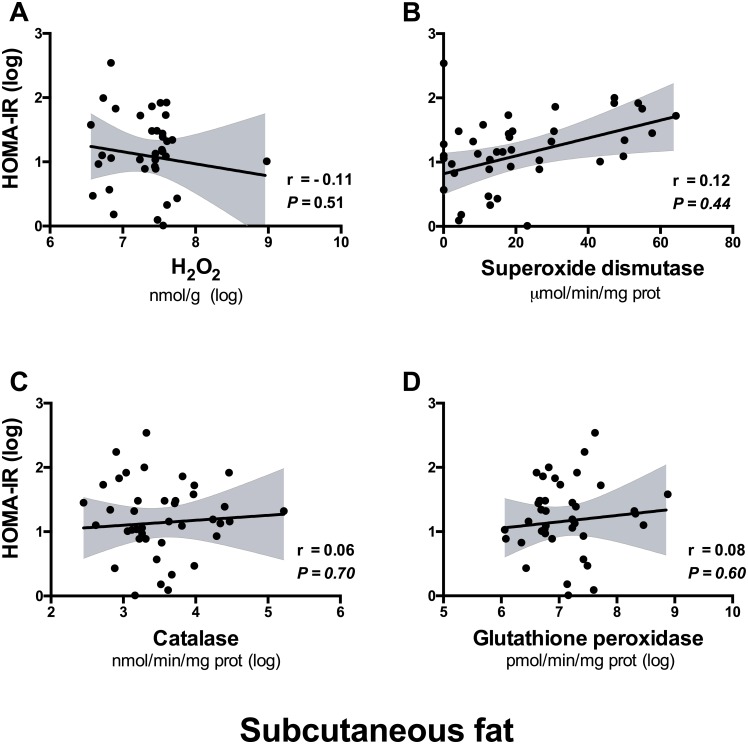
*A*, *B*, *C*, *D* Lack of associations of H_2_O_2_ *(A)* or activity of its metabolizing enzymes *(B-D)* in subcutaneous adipose tissue with the homeostasis model of insulin resistance (HOMA-IR) in the total population (N = 43). Each scatter plot shows the best-fit linear regression line and 95% confidence intervals, with Pearson correlation coefficients. All parameters are log-transformed apart from superoxide dismutase activity.

In line with the association of visceral fat H_2_O_2_ metabolism with central adiposity, positive correlations were observed between H_2_O_2_ concentrations in visceral fat and serum insulin (r = 0.42, p = 0.016; Table 3) and HOMA-IR (Fig 2A), but not serum glucose (Table 3). Visceral adipose tissue superoxide dismutase and catalase activities were also positively associated with HOMA-IR (Fig 2B–2D), and catalase additionally correlated with fasting glucose (r = 0.38, P = 0.029; Table 3). In contrast, glutathione peroxidase activity in visceral fat was unrelated to insulin sensitivity. Further, no associations were observed between H_2_O_2_ and its metabolizing enzymes in the subcutaneous fat depot and insulin sensitivity (Fig 3A–3D).

Given the particularly strong association of visceral fat catalase activity with insulin resistance (r = 0.52, P = 0.002; Fig 2C) we used multivariate linear regression to investigate whether catalase was an independent predictor of HOMA-IR, after adjustment for its major determinant (BMI) [31], and age. We fitted similar models separately for visceral fat superoxide dismutase activity and H_2_O_2_ concentrations. Catalase activity in visceral fat was an independent determinant of HOMA-IR, explaining ~18% of the variance (*ß* = 0.42, P = 0.016; Table 4), after adjustment for age and BMI. The association of catalase with HOMA-IR persisted after additional adjustment for smoking and LDL-cholesterol concentrations ((*ß* = 0.52, P = 0.006).

**Table 4:** Visceral adipose tissue parameters as independent predictors of HOMA-IR ^1^.

Model		Standardized coefficient *(β)*	*P value*
**1**	**Age**	-0.14	*0*.*37*
	**BMI**	***0*.*34***	***0*.*044***
	**Catalase** ^2^	**0.42**	***0*.*016***
**2**	**Age**	-0.09	*0*.*58*
	**BMI**	**0.42**	***0*.*013***
	**SOD**^2^	0.30	*0*.*074*
**3**	**Age**	0.04	*0*.*80*
	**BMI**	**0.43**	***0*.*021***
	**H**_**2**_**O**_**2**_	0.26	*0*.*15*

^(1)^ Using multiple linear regression with HOMA-IR (log-transformed) as the independent variable. N = 33. The predictor variables included simultaneously in each model are those for which results are shown.

^(2)^ Log-transformed variables

## Discussion

Animal studies demonstrate that genetic deficiency of the H_2_O_2_-scavenging enzyme catalase triggers adipose tissue oxidative stress, deterioration of insulin sensitivity and susceptibility to diabetes [32, 33]. In obese wild-type mice, H_2_O_2_ production is increased in adipose tissue, and pharmacologic lowering of H_2_O_2_ production improved insulin sensitivity [12]. The present study explores the relevance of these findings to humans. Men with central obesity featured increased superoxide dismutase activity, and accumulation of its product, H_2_O_2_, in visceral, but not subcutaneous fat. However, catalase activity was increased, rather than deficient, in visceral fat from obese men, and both H_2_O_2_ and catalase correlated positively with HOMA-IR. These findings suggest that adipose tissue catalase shows compensatory up-regulation in response to obesity-induced H_2_O_2_ accumulation, and that oxidative stress in visceral fat is linked to insulin resistance in obese humans.

A main finding was the accumulation of H_2_O_2_ in visceral fat in men with abdominal obesity. H_2_O_2_ has long been recognized to induce insulin resistance in 3T3-L1 adipocytes upon prolonged, high dose application [34]. Subsequent studies showed that applying 2 potentially diabetogenic substances to 3T3-L1 adipocytes triggered oxidative stress that preceded the occurrence of insulin resistance [35]. Furthermore, insulin resistance was prevented by antioxidant treatment, demonstrating a causal role for ROS in deterioration of insulin sensitivity [35]. Studies in animals support in vitro findings by showing that obese mice feature increased H_2_O_2_ production in parametrial visceral fat by NADPH oxidase-4 (NOX4) [12], and that pharmacologic inhibition of H_2_O_2_ production improved insulin sensitivity [12]. Whether visceral or subcutaneous adipose tissue in humans is similarly implicated in the oxidative stress underlying insulin resistance was unclear. The present study shows that the visceral fat depot features oxidative stress in human obesity that correlates with peripheral insulin resistance. Enhanced H_2_O_2_ production has also been shown to occur in skeletal muscle of men fed a high-fat diet [13], and of obese, insulin-resistant women with polycystic ovary syndrome [36]. *In vitro*, H_2_O_2_ exposure impairs insulin signaling by various mechanisms including suppression of insulin-induced Akt phosphorylation, and inhibition of both transcription of glucose transporter 4 (GLUT 4) and its translocation to the cell membrane [37, 38].

H_2_O_2_ is catabolized by several pathways, including degradation by catalase to release O_2_ and water, and reduction by glutathione peroxidase, with consumption of reduced glutathione. While genetic catalase deficiency is associated with diabetes in humans [39] and animals [33], our data suggests that in the general population, catalase activity is increased, rather than deficient, in adipose tissue of insulin resistant subjects. A high fat meal was found to increase plasma catalase activity by 30% in obese humans [40]. Further, an average weight loss of 9.5 kg in overweight and obese subjects induced a 40% reduction in adipose tissue catalase levels [41]. The decrease in catalase was associated with enhanced adipose tissue GLUT 4 translocation and improved insulin sensitivity [41]. These studies, together with our data, suggest that insulin resistant states go hand-in hand with catalase up-regulation.

In contradiction to our findings, Amirkhizi et al observed decreased erythrocyte catalase activity in women with abdominal obesity [42]. The discrepancy may be related to the different genders, or differences in participant metabolic characteristics or the tissues studied. We observed strong positive correlations of catalase activity with waist circumference and HOMA-IR in men. Upregulation of catalase activity has also been reported in serum [43, 44] and erythrocytes [45] from subjects with diabetes. Among several oxidative stress markers, serum catalase activity was the best correlate of poor glycemic control [43]. Our data is also consistent with animal studies showing immediate and sustained upregulation of catalase in cardiac muscle from mice fed a high-fat diet [46]. In rats fed a high-fat diet, an increase in visceral fat accumulation and insulinemia were associated with enhanced catalase activity in liver [47], while amelioration of these features by pioglitazone decreased catalase activity [47]. Despite the heterogeneity of the models and tissues investigated, collective evidence suggests that obesity-induced oxidative stress may not result from catalase deficiency, and is often, though not consistently [42], associated with catalase up-regulation.

The mechanism of increased catalase activity in our Ow/Ob group is unclear, but some clues can be derived from the literature. Catalase activity increases in response to increasing H_2_O_2_ during differentiation of human [48] as well as 3T3-L1 [49] preadipocytes. It is thus conceivable that catalase activity may increase in response to the increased H_2_O_2_ that accompanies adipose tissue expansion in obesity, as observed in visceral fat in the present study. Catalase activity in human plasma has also been shown to consistently increase in response to insulin infusion [50]. If adipose tissue catalase is similarly responsive to circulating insulin concentrations, this might contribute to the catalase induction in Ow/Ob subjects, who had 47% higher plasma insulin compared to lean subjects. It would also explain the strong positive association of visceral fat catalase activity with insulin, and with HOMA-IR, independent of BMI. The purpose of this adipose tissue catalase upregulation in obesity is not conclusively known, but, at least in animal models, adipose tissue catalase plays an important role not only in antioxidant defense but also in protection against obesity-associated inflammation [32].

Notably, glutathione peroxidase activity was not altered in visceral or subcutaneous fat from centrally obese, insulin resistant individuals, despite a 45% increase in visceral fat H_2_O_2_. A similar lack of induction of glutathione peroxidase was observed in cardiomyocytes from high-fat fed mice [46], and in erythrocytes from patients with diabetes [45]. It is postulated that the lower binding affinity of catalase for H_2_O_2_, compared to glutathione peroxidase, renders catalase ideal for scavenging toxic levels of H_2_O_2_, and hence explains its induction in response to gross increases in H_2_O_2_ [46]. Consistent with this, *in vitro* studies of human erythrocytes demonstrated a linear increase of catalase activity with increasing H_2_O_2_, while glutathione peroxidase showed early saturation at nanomolar H_2_O_2_ concentrations [51].

### Strengths and limitations

The present study is primarily limited by the cross-sectional design, which makes it impossible to ascertain the sequence of events in the obesity-oxidative stress-insulin resistance paradigm. Based on animal studies [12], and human interventional studies in muscle [13], we interpreted our data to indicate that obesity increases adipose tissue H_2_O_2_ generation, which in turn promotes insulin resistance. However, there is ample literature evidence suggesting reverse causality, e.g. that insulin resistance itself increases oxidative stress [52], and that oxidative stress may be causal in obesity as shown by enhanced adipogenesis by ROS [53].

The study benefits from several strengths, including selecting a homogeneous population of sedentary non-diabetic men, thus avoiding confounding by gender, exercise, menopausal state and diabetes which influence redox state [43, 44, 54]. However, this limits generalizability of the data to women. Another advantage is that both subcutaneous and visceral fat samples were available from subjects over a wide BMI range, encompassing normal-weight, overweight and moderate obesity. This range is more relevant from a population perspective than the more commonly studied samples from severely obese bariatric surgery patients.

## Conclusion

In conclusion, men with abdominal obesity featured superoxide dismutase induction, and H_2_O_2_ accumulation in the visceral fat depot, despite upregulation of catalase activity. H_2_O_2_ concentrations, and superoxide dismutase and catalase activities in visceral fat correlated positively with HOMA-IR. This data in humans supports animal studies implicating dysregulated adipose tissue H_2_O_2_ metabolism in peripheral insulin resistance.

## Supporting information

S1 File Table 1. Obesity-related measures and adipose tissue H_2_O_2_ metabolism in smokers and non-smokers. (DOCX)

S2 File Table 2. Comparison of H_2_O_2_ concentration and activity of its metabolizing enzymes in the subcutaneous versus visceral fat compartments. (DOCX)
