# Supplementary material for: Perturbed adipose tissue hydrogen peroxide metabolism in centrally obese men: Association with insulin resistance
Source: PLoS One. 2017 May 18;12(5):e0177268. doi: 10.1371/journal.pone.0177268 (PMC5436683; doi:10.1371/journal.pone.0177268)
Supplement: S1 File — (DOCX) [file pone.0177268.s001.docx]

**Table 1. Obesity-related measures and adipose tissue hydrogen peroxide metabolism in smokers and non-smokers**

|  | **Non-smokers**  **N = 19** | **Smokers**  **N = 21** | ***P value*** |
| --- | --- | --- | --- |
|  |  |  |  |
| **BMI (**Kg/m^2^) | 25.9 (21.3-28) | 25.1 (23.0-28.3) | *0.61* |
| **Glucose** (mg/dl) | 106 (95-124) | 120 (105-127) | *0.16* |
| **Insulin** (mU/L) | 12.0 (9.3-17.3) | 10.1 (8.5-19.1) | *0.56* |
| **HOMA-IR** | 3.75 (2.44-4.84) | 3.10 (2.63-4.40) | *0.90* |
| ***Visceral fat parameters*** |  |  |  |
| **H_2_O_2_**  (mmol/g tissue) | 0.84 (0.69-1.01) | 0.79 (0.60-0.90) | *0.26* |
| **Superoxide dismutase**  (μmol/ min/ mg protein) | 20.9 (9.10-38.8) | 25.5 (15.5-50.8) | *0.16* |
| **Catalase**  (nmol/ min/ mg protein) | 47.1 (31.5-83.2) | 34.4 (23.9-53.6) | *0.51* |
| **Glutathione peroxidase**  (nmol/ min/ mg protein) | 1.28 (0.84-1.62) | 1.05 (0.89-1.74) | *0.97* |
| ***Subcutaneous fat parameters*** |  |  |  |
| **H_2_O_2_**  (mmol/g tissue) | 1.63 (0.93-1.87) | 1.74 (1.39-1.91) | *0.37* |
| **Superoxide dismutase**  (μmol/ min/ mg protein) | 18.5 (12.9-30.9) | 18.6 (11.3-44.6) | *0.69* |
| **Catalase**  (nmol/ min/ mg protein) | 27.4 (22.6-41.1) | 31.6 (22.3-53.5) | *0.98* |
| **Glutathione peroxidase**  (nmol/ min/ mg protein) | 1.02 (0.79-1.39) | 1.36 (0.83-1.89) | *0.29* |

Data are presented as median (25^th^, 75^th^ percentiles), and groups compared by Mann Whitney *U* test. N = 43 for serum and subcutaneous fat parameters, and N = 33 for visceral fat parameters.
