# Supplementary material for: Perturbed adipose tissue hydrogen peroxide metabolism in centrally obese men: Association with insulin resistance
Source: PLoS One. 2017 May 18;12(5):e0177268. doi: 10.1371/journal.pone.0177268 (PMC5436683; doi:10.1371/journal.pone.0177268)
Supplement: S2 File — (DOCX) [file pone.0177268.s002.docx]

**Table 2. Comparison of H_2_O_2_ concentration and activity of its metabolizing enzymes in the subcutaneous versus visceral fat compartments**

|  | H_2_O_2_ | Superoxide dismutase | Catalase | Glutathione peroxidase |
| --- | --- | --- | --- | --- |
| **Subcutaneous/**  **Visceral (%)** | 212 | 74.2 | 70.6 | 96.4 |
| ***P value^1^*** | *<0.001* | *0.14* | *0.059* | *0.93* |

(1) Wilcoxon Signed Rank Test. N = 33.
